# Electricity in Dental Practice

**Published:** 1884-03

**Authors:** 


### ELECTRICITY IN DENTAL PRACTICE.

There is no field in economic science that offers to the student such inviting opportunities as the study of the conservation of force. In view of the later demonstrations that all force is a unit, differing only in its mode of manifestation, and that chemical action, electricity, heat, light, etc., are in reality identical and mutually interchangeable the one into the other, that wherever there are molecular or other changes or motions in matter there some one of the different manifestations of force is generated or eliminated, it is readily seen that incalculable units of energy are daily going to waste, that should be utilized for the benefit of man. Every wave of the sea, all the currents of the rivers, the ebb and flow of the tides, the winds that blow, are but molecular changes generating force that, if properly economized, would be far in excess of all the wants of man. The question has doubtless arisen in every thoughtful mind, whence shall future generations derive the necessary heat and light when the coal fields shall have been exhausted? for it is but a question of time when the last ton of coal shall have been mined. It is only necessary to remember that in the flow of the Hudson River, sufficient of energy is evolved to satisfy the demands of all the cities that may hereafter exist upon its banks. Could that force be economized and transferred into electricity, and that into light and heat as might be demanded, the great problem of the future would be solved.

At present we comprehend but dimly the nature and character of that manifestation of force that we call electricity, but already its possibilities are shadowed forth. Numerous attempts have been made to employ it as a motive power in running dental and other machinery, but so far its application has been at such a tremendous disadvantage, through our ignorance of its real character, and the wastage of energy has been so out of proportion to the amount generated, that it can scarcely be considered a practical success. Great advances have been made in the past five years, but we are still a long way from the desired end. It would appear that its direct application to most machinery as a motive power is impracticable, and students in electrical science might profitably consider if it cannot be transformed into some of the other manifestations of force, and thus be more readily employed. By changing it into heat, in the electro-cautery, a very valuable appliance has been added to surgery, especially in operations in the mouth, where effusion of blood is so embarrassing to the operator.

But it is of its conversion into light that we desire most particularly to speak. So pure and so intense are the rays derived from it that, even with the imperfect apparatus already devised, a sight of the oral tissues under its illumination is a new revelation to the dentist. It is not long since that, in conversation with a prominent New York dentist who uses it, in relating a case of pericementitis, the origin of which was obscure, he remarked that upon placing the electric light in the mouth he *saw* that the root of the tooth was not filled to the apex. "What are you giving me?" was the comment. He repeated his assertion, only to be met with another exclamation of incredulity. "I am not in the habit of making statements that I cannot substantiate," said he; "come with me into the operating room." Calling his assistant, he placed the light in his mouth, and we were astonished at the result. We do not mean to assert that the teeth and their investing tissues were made transparent, but we do say that so intense was the illumination that the condition of the tissues could be, by the experienced operator, very clearly observed. The congestion produced by pressure altered the appearance of the soft tissues, so that even though it were comparatively deep-seated, it was quite plainly indicated. An inflamed pulp changed the looks of a tooth, interrupting the semi-translucency. A filling could be plainly seen through a tooth, and a filled root presented a different appearance from one that was unfilled. But it was especially in determining the condition of proximate surfaces in close contact, that we were impressed with the usefulness of the electric light. If such surfaces were rough and unpolished, if food habitually lodged between them, the fact was at once apparent. If softening of the enamel had begun, the premonitory symptom of approaching decay, it was instantaneously revealed. If a pocket had formed beneath the gum, and foreign matter was lodged there, it needed not the probe to determine the fact. Incipient pyorrhea could be immediately detected. In short, we were so impressed with the manifest advantages to be obtained by the use of the electric light in the oral cavity, that in our mind's eye we saw the solution of a problem that has long vexed our practice.

The apparatus that our friend exhibited to us was the best of the many that we had inspected, in both Europe and America, but it was by no means perfect. We are waiting until something a little better shall have been offered, and then we expect that the electric light will be an essential in our operating room.

One great objection to the employment of electricity by dentists, is the constant care necessary to keep a battery in complete order. The busy practitioner has not the time to spend in keeping plates clean, and the fluids in proper condition, while few are blessed with assistants to whom this duty can with confidence be entrusted. The Trouvé-Faure storage batteries promise good results, but they are far from being perfect at present. The wastage is so great, the conservation of the electric force is so imperfect, that they are very unsatisfactory at best. But the principle of their construction is so clearly in harmony with what we know of the evolution of force, that we may hope for great things from it in the future. It is a misunderstanding to suppose that it is *electricity* that is stored. In reality it is *chemical* force, which, being set free, results in the elimination of electricity. In other words, in charging such a battery electricity is changed into chemical energy, which in action is re-converted into electricity. But it will readily be understood that in these changes very much of the force is wasted, to say nothing of the constant *leakage* of energy which takes place when the battery is not in action. When the constant study and experiments of electrical experts shall have taught how to economize better, we may confidently look to see electricity employed to advantage in the office of every operative dentist.

